# Skin Metastasis From Solid Tumors: Is Targeted Therapy Making an Impact?

**DOI:** 10.7759/cureus.74362

**Published:** 2024-11-24

**Authors:** Mohamed Arshad, Ashwin J Paul, Raghul Kumar

**Affiliations:** 1 Medical Oncology, Madras Medical College, Chennai, IND; 2 Medical Oncology, Government Arignar Anna Memorial Cancer Hospital, Kanchipuram, IND; 3 Dermatology, Madras Medical College, Chennai, IND

**Keywords:** breast carcinoma skin metastasis, geftinib, lung cancer, palbociclib, targeted therapeutics

## Abstract

Cutaneous metastasis from solid tumors is a rare manifestation that presents in the clinic. It is usually indicative of poor prognosis, with a poor response to conventional chemotherapy. The introduction of targeted therapy has changed the treatment paradigm in oncology, improving the quality of life and survival of all cancer patients. Here, we present two cases of solid tumors with skin metastases that had a good response to targeted systemic therapy. This case report also discusses the various modes of presentation and the therapeutic options for metastatic cutaneous lesions from solid tumors.

## Introduction

Cancer is one of the leading causes of morbidity and mortality in India. The estimated number of incident cancer cases in India for the year 2022 exceeded 14 million. In India, one in nine people is likely to develop cancer in their lifetime. The leading cancer sites are breast, head and neck, cervix, and lung [1]. The skin is one of the metastatic sites for these solid tumors. It is a relatively uncommon phenomenon, with the reported incidence ranging from 0.7% to 10.4% among various case series [2]. As skin metastases can be suspected and detected earlier compared to metastases in other organs, clinicians should be aware of the various appearances of such lesions, and pathologists should recognize the different patterns of metastatic deposits in the skin. The biopsy evaluation of such deposits often yields information about the probable site of the primary tumor based on the histological appearance of the tumor deposits. This information can be further refined by using histochemical stains and immunohistochemical studies on the biopsy sections [3-7]. Accurate recognition of cutaneous metastases on biopsy examination, especially in cases of unknown primary tumors, initiates relevant clinical and radiological investigations to confirm the site and type of the primary neoplasm.

## Case presentation

Case 1

**Figure 1:**
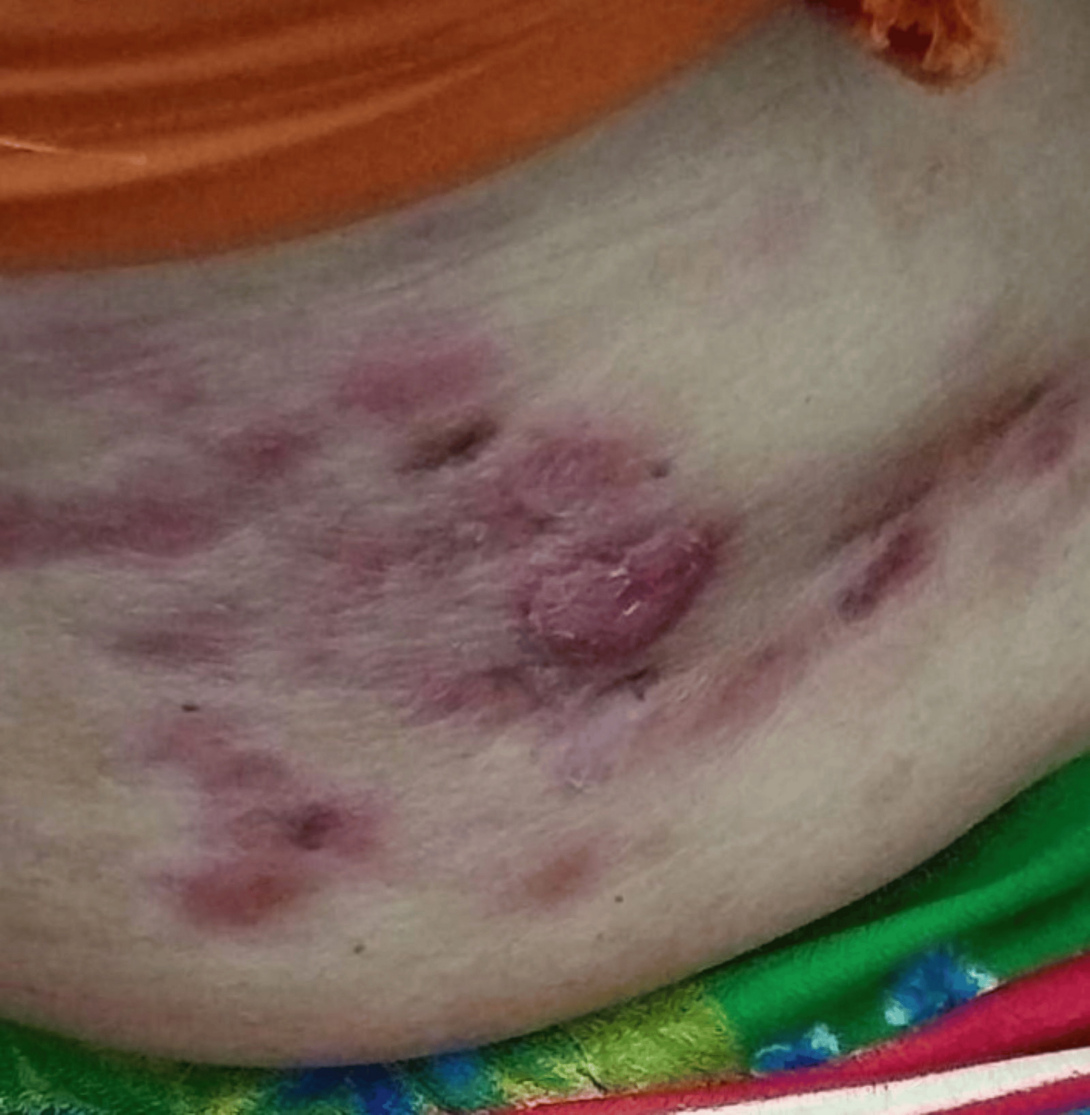
Erythematous nodular skin lesions of the breast cancer patient before targeted therapy

A 46-year-old postmenopausal female was diagnosed with carcinoma of the right breast, pathological stage pT3N1M0. Immunohistochemistry (IHC) was positive for both hormone receptors, estrogen receptor/progesterone receptor (ER/PR), and negative for HER2. She was treated with a right-modified radical mastectomy, followed by adjuvant chemotherapy and post-mastectomy radiation therapy (PMRT). She received four cycles of anthracycline and cyclophosphamide, followed by four cycles of paclitaxel as adjuvant chemotherapy. Additionally, she was given five years of tamoxifen as adjuvant hormonal therapy. After one year of follow-up, she presented with nodular and patchy erythematous lesions over the waist (Figure 1), abdomen, and chest region. Examination of the opposite breast was normal; there was no axillary lymphadenopathy, and systemic examination was unremarkable; clinically, there was no evidence of metastasis.

**Figure 2:**
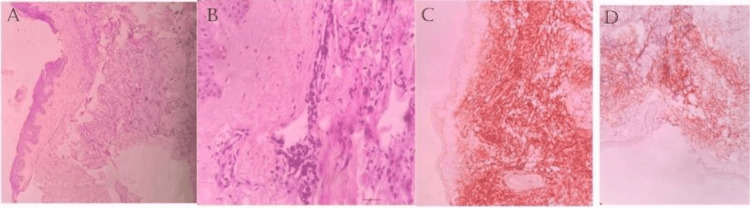
A) microscopic examination at 10x magnification, B) microscopic examination at 40x magnification of the skin lesion showing malignant neoplasm arranged in cords, tubules and clusters, C) immunohistochemical staining of the lesion demonstrating cytoplasmic staining for mammaglobin, D) immunohistochemical staining of the skin lesion demonstrating surface expression of ER over the tumor cells.

Biopsy taken from the skin lesion showed malignant neoplastic cells arranged in cords, tubules, and clusters (Figures 2A, 2B). Further, IHC examination of skin biopsy was positive for mammoglobin (Figure 2C) and ER (Figure 2D). 

**Figure 3:**
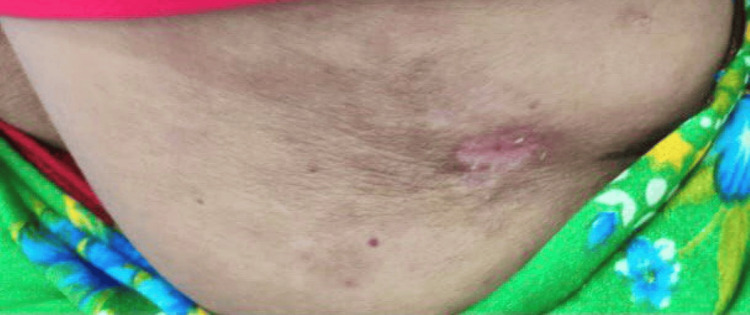
Resolved skin lesion after initiation of palbociclib and letrozole

The patient's PET CT showed no other sites of metastasis. She was treated with palbociclib and letrozole. After three months of treatment, all her lesions disappeared, as shown in Figure 3.

Case 2

**Figure 4:**
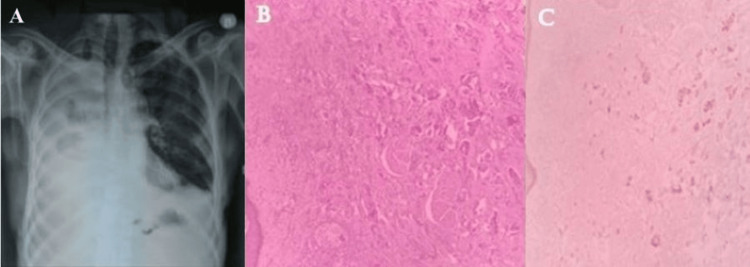
A) chest X-ray of the patient revealing right sided massive pleural effusion, B) biopsy from the skin lesion demonstrating malignant neoplasm arranged in clustered and nests, C) immunohistochemical staining for thyroid transcription factor (TTF) was positive in few of the tumor cells.

A 70-year-old male, a non-smoker with no comorbidities, presented with chronic cough and subacute dyspnea. He had a history of weight loss and loss of appetite. A chest X-ray (Figure 4A) showed a right-sided massive pleural effusion, and cytology was positive for malignancy. Pleural fluid cell block confirmed metastatic adenocarcinoma from a lung primary (Figure 4B). The patient was diagnosed with stage IV-A adenocarcinoma of the lung.

**Figure 5:**
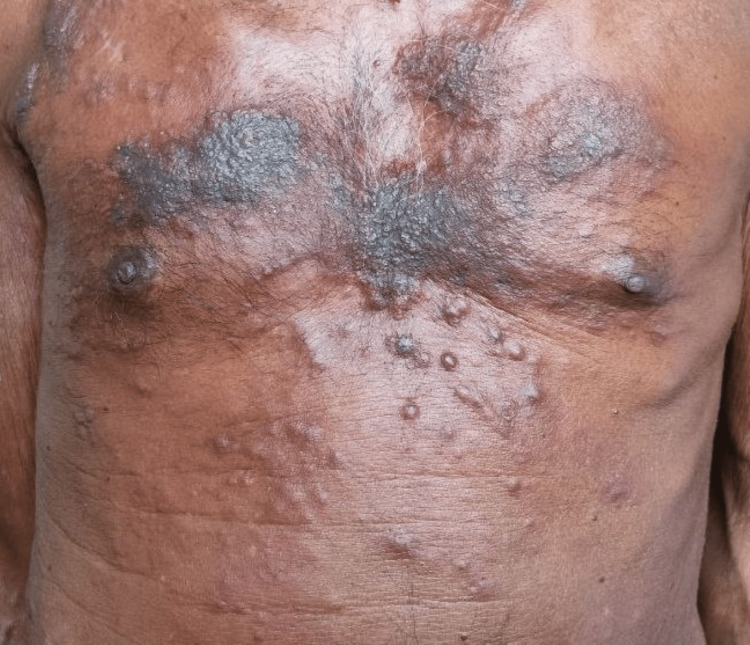
Skin lesion of the patient showing multiple violaceous , erythematous skin lesions over his chest wall.

He was started on palliative chemotherapy with carboplatin and pemetrexed. He had a partial response after four cycles of chemotherapy, so he continued on maintenance pemetrexed for six cycles. Subsequently, he presented with multiple violaceous, erythematous skin lesions over his chest wall (Figure 5). A biopsy confirmed metastatic carcinomatous deposits from the lung, with IHC positive for TTF (thyroid transcription factor), as shown in Figure 4C. Further molecular testing was positive for the Epidermal growth factor receptor (EGFR) L858R mutation. He was started on gefitinib 250 mg once a day.

**Figure 6:**
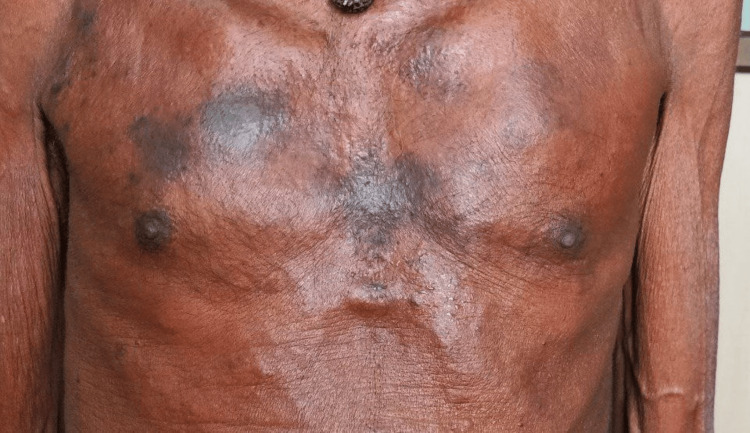
Resolving skin lesion after starting the patient on EGFR inhibitor.

After starting the patient on the EGFR inhibitor gefitinib, the patient had a good response, and all the skin lesions started resolving, as shown in Figure 6.

## Discussion

Skin metastases from solid tumors are rare in clinical practice. They mostly present as recurrent lesions but, in exceptional cases, may occur concurrently or even prior to the detection of the primary tumor. Cutaneous metastases can take various forms, including plaques, nodules, papules, and vesicular lesions, with nodules being the most common. The pathogenesis involves six stages: local invasion, intravasation, survival in circulation, arrest in a distant organ, extravasation, and micrometastasis formation [8]. In females, the primary tumors most frequently associated with skin metastasis are breast (63.19%), large intestine (10.41%), and lung (4.16%). In men, the primary sites are the lung (33.84%), stomach (12.3%), and larynx (7.69%) [9]. The most common areas affected are the anterior thorax, abdomen, head and neck, and limbs [10].

Most cutaneous metastases occur near the primary tumor and typically present as firm, round, or oval, mobile, non-painful nodules of varying size, which can sometimes ulcerate. Specific presentations include:

Carcinoma erysipeloides, which is a sharply demarcated red patch due to the spread of cancer blocking lymphatic vessels in adjacent skin; en cuirasse or sclerodermoid carcinoma, which includes indurated, scar-like plaques due to cancer cell infiltration into collagen; carcinoma telangiectoides includes red patches with multiple blood vessels (telangiectases) or lymphatic vessels (lymphangioma-like) [11].

Diagnosis primarily relies on the patient’s cancer history and biopsy. IHC assists in distinguishing primary skin malignancy from metastatic lesions and in identifying the primary tumor site. There are no published protocols or approved guidelines for the management of metastatic skin lesions. Skin-directed therapies, which had limited success (less than 10%) with conventional chemotherapy, include options such as surgical excision, radiation, intralesional chemotherapy, imiquimod cream, cryotherapy, and various laser therapies [12]. The advent of targeted therapies and precision oncology has increased the response rates to treatment, providing better survival and quality of life compared to conventional chemotherapy.

Palbociclib, a cyclin-dependent kinases 4/6 (CDK4/6) inhibitor that induces G1 phase cell cycle arrest, is used in combination with aromatase inhibitors to treat metastatic luminal breast cancer, achieving an overall response rate of 42.1%. In the phase 3 randomized trial comparing palbociclib plus letrozole versus letrozole plus placebo in the management of metastatic luminal breast cancer, the patients randomized to palbociclib plus letrozole demonstrated a median PFS of 24.8 months [13]. On the other hand, the median PFS for chemotherapy like capecitabine, eribulin, and vinorelbine is less than 12 months. In our first case, the patient achieved a complete response with palbociclib and letrozole, maintaining this response for 24 months.

Gefitinib, a first-generation EGFR tyrosine kinase inhibitor, is effective in EGFR-mutated metastatic lung adenocarcinoma with an overall response rate of 73.7%, compared to 30.7% for platinum-based chemotherapy. In the phase 3 randomized trial of gefitinib versus chemotherapy in the management of metastatic EGFR mutated lung cancer, patients randomized to the gefitinib arm had a median progression-free survival (PFS) of 10.8 months, and patients randomized to the chemotherapy arm had a median PFS of only 5.4 months demonstrating the superiority of gefitinib to chemotherapy in the management of metastatic EGFR mutated lung cancer [14]. The second patient achieved a complete response, with all skin lesions resolving within two months of gefitinib treatment, and maintained this response for 13 months.

Both patients had complete resolution of their skin lesions using systemic targeted therapy without the need for local treatments such as imiquimod cream or laser therapy. Our case report demonstrates the efficacy of targeted therapy in the management of metastatic skin lesions. Yet, randomized controlled trials are required to demonstrate the superiority of targeted systemic therapy compared to chemotherapy plus local skin-directed therapy.

## Conclusions

The advantages of targeted cancer drugs include that they are mostly administered orally and have minimal side effects compared to chemotherapy. Additionally, they act on both the primary tumor and skin lesions, thereby reducing the need for skin-directed therapy. Although metastatic cancer has a high mortality rate, these targeted systemic agents have helped patients live longer and with a better quality of life, providing higher and more durable responses compared to conventional chemotherapy.
